# Na^+^-induced Ca^2+^ influx through reverse mode of Na^+^-Ca^2+^ exchanger in mouse ventricular cardiomyocyte

**DOI:** 10.18632/oncotarget.4878

**Published:** 2015-08-06

**Authors:** Zhen-Yu Yan, Tao Ban, Yao Fan, Wei-Ran Chen, Hong-Li Sun, Hanying Chen, Quo-Fen Qiao, Bai-Yan Li

**Affiliations:** ^1^ Department of Pharmacology, Harbin Medical University, Harbin, China; ^2^ Riley Heart Research Center, Division of Pediatric Cardiology, Herman B. Wells Center for Pediatric Research, Department of Pediatrics, Indiana University School of Medicine, Indianapolis, USA; ^3^ Key Laboratory of Cardiovascular Medicine Research, Harbin Medical University, Harbin, China; ^4^ Department of Pharmacology, Da-Qing Campus of Harbin Medical University, Da-Qing, China

**Keywords:** Pathology Section, reverse mode of Na^+^-Ca^2+^ exchanger, dobutamine, action potential, voltage-gated ion channel, ventricular cardiomyocyte

## Abstract

**Background:**

Dobutamine is commonly used for clinical management of heart failure and its pharmacological effects have long been investigated as inotropics via β–receptor activation. However, there is no electrophysiological evidence if dobutamine contributes inotropic action due at least partially to the reverse mode of Na^+^-Ca^2+^ exchanger (NCX) activation.

**Methods:**

Action potential (AP), voltage-gated Na^+^ (*I*_Na_), Ca^2+^ (*I*_Ca_), and K^+^ (*I*_to_ and *I*_K1_) currents were observed using whole-cell patch technique before and after dobutamine in ventricular cardiomyocytes isolated from adult mouse hearts. Another sets of observation were also performed with Kb-r7943 or in the solution without [Ca^2+^]_o_.

**Results:**

Dobutamine (0.1–1.0 μM) significantly enhanced the AP depolarization with prolongation of AP duration (APD) in a concentration-dependent fashion. The density of *I*_Na_was also increased concentration-dependently without alternation of voltage-dependent steady-status of activation and inactivation, reactivation as well. Whereas, the activities for *I*_Ca_, *I*_to_, and *I*_K1_ were not changed by dobutamine. Intriguingly, the dobutamine-mediated changes in AP repolarization were abolished by 3 μM Kb-r7943 pretreatment or by simply removing [Ca^2+^]_o_ without affecting accelerated depolarization. Additionally, the ratio of APD_50_/APD_90_ was not significantly altered in the presence of dobutamine, implying that effective refractory period was remain unchanged.

**Conclusion:**

This novel finding provides evidence that dobutamine upregulates of voltage-gated Na^+^ channel function and Na^+^ influx-induced activation of the reverse mode of NCX, suggesting that dobutamine may not only accelerate ventricular contraction via fast depolarization but also cause Ca^2+^ influx, which contributes its positive inotropic effect synergistically with β-receptor activation without increasing the arrhythmogenetic risk.

## INTRODUCTION

Dobutamine is a sympathomimetic drug that has long been used in the treatment of heart failure [1] and cardiogenic shock [2] on the basis of its positive inotropic action. Its primary mechanism is direct stimulation of β-receptors of the sympathetic nervous system coupled with intracellular Ca^2+^ mobilization through G-protein-couple receptor and cyclic adenosine-monphosphate (cAMP) pathway. However, if other potential mechanism except for β-receptor stimulation involved in dobutamine-mediated intracellular Ca^2+^ mobilization is not fully understand so far. Recent finding has shown that Na^+^ influx during the early phase of action potential (AP) induces an intracellular Ca^2+^ increase through activation of the reverse mode of Na^+^-Ca^2+^ exchanger (NCX) [3–5] and there is no such report in the literatures if dobutamine share the similar mechanisms. Therefore, this study was designed to answer the following questions regarding the electrophysiological profiles of dobutamine: [1] if voltage-gated Na^+^ channel is activated; [2] if Na^+^ influx induces the Ca^2+^ entry via the reverse mode of NCX in an extracellular-dependent manner; and [3] the relation between [1] and [2]. To experimentally verify these questions, both AP and voltage-gated ion channel currents (*I*_Na_, *I*_Ca_, *I*_K1_, and *I*_to_) were investigated on ventricular cardiomyocytes isolated from adult mouse heart, respectively, using whole-cell patch technique [6, 7] before and after administration of dobutamine. Additionally, separate sets of current-clamp observations were also performed by pretreatment of cardiomyocytes with Kb-r7943, selective NCX blocker, with normal Tyrode's solution or calcium free Tyrode's solution to verify the involvement of NCX and Ca^2+^-dependency. This observation provides a solid evidence to suggest that dobutamine mediates Na^+^ influx through voltage-gated Na^+^ channel during AP depolarization and consequently induces an extracellular Ca^2+^-dependent Ca^2+^ influx through the reverse mode of NCX in ventricular cardiomyocyte of adult mouse heart.

## MATERIALS AND METHODS

### Animals

For consistency, only 8 week-old young adult male mice were used for experiments unless otherwise indicated. All animal protocols were approved by Harbin Medical University or Indiana University School of Medicine Institutional Animal Care and Research Advisory Committee.

### Chemical agents

Dobutamine and Kb-r7943 [8], a selective blocker for the reverse mode of Na^+^-Ca^2+^ exchanger (NCX), were ordered directly from Sigma-Aldaich. Chemical agents for cell culture, enzymatic isolation of cardiomyocyte, and recording solutions were purchased from the regular commercial sources.

### Ventricular cardiomyocyte isolation

**Figure 1:**
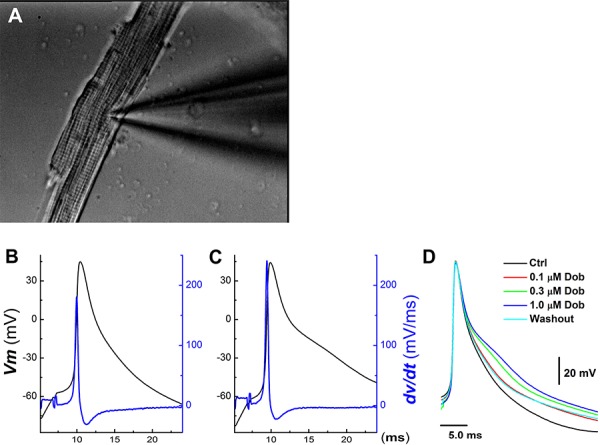
Concentration-dependent effects of dobutamine on action potential (AP) discharge character in ventricular cardiomyocyte isolated from adult mouse heart AP was elicited from the resting membrane potential by a brief pulse with 2 ms duration using current-clamp mode. **A.** representative image of ventricular cardiomyocyte with recording pipette collected during recording under 40× light microscope; **B.** and **C.** representative AP and derivative over the membrane potential before and after 1 μM dobutamine, respectively; **D.** superimposition of APs recorded in the absence and presence of 0.1 – 1 μM dobutamine.

Adult mouse single ventricular cardiomyocytes were isolated using Langendorff-perfused mouse hearts and standard enzymatic techniques as previously reported with minor modifications [9]. Briefly, mice were heparinized and sacrificed by cervical dislocation. The hearts were rapidly removed and retrogradely perfused through the aorta using a modified Langendorff apparatus. The preparation was perfused with calcium free Tyrode's solution (in mM: NaCl 126, KCl 5.4, HEPES 10, NaH_2_PO_4_ 0.33, MgCl_2_ 1.0, taurine 10, glucose 10, and pH adjusted to 7.4 with 1.0 N NaOH) for 5 min, and then switched to digestive solution (calcium free Tyrode's solution containing Collagenase type-II 0.4 mg/ml, protease 0.02 mg/ml and BSA 1.0 mg/ml). Left atria and ventricular tissue were collected and titrated gently in calcium free Tyrode's solution containing 0.5% BSA to obtain single cells. All solutions were gassed with 95% oxygen and 5% carbon dioxide. Single rod-shaped and Ca^2+^ tolerant cells with clear cross-striations (Figure 1A) were used for electrophysiological investigation.

### Electrophysiology

Both current- (AP) and voltage-clamp recordings (*I*_Na_, *I*_Ca_, *I*_k1_, and *I*_to_) were conducted using standard whole-cell patch-clamp techniques [10, 11] with an Axopatch 700B amplifier (Axon Instruments). Briefly, The recording electrodes (Borosilicate glass, Sutter) were pulled (P-97, Sutter Instruments) and polished (F-83, Narishige) down to 1.2 – 1.8 MΩ when filled with pipette solution. After formation of the gigaohm-seal, the capacitance was electronically compensated and the cell membrane under the pipette tip was then ruptured by a brief increase in suction, forming the whole-cell recording configuration. After 2–5 min period for intracellular dialysis, the Tyrode solution was changed by bath perfusion of extracellular recording solution designed for *I*_Na_, *I*_Ca_, *I*_k1_, and *I*_to_ recordings, respectively. All cells were recorded at room temperature (22 – 23°C). Current amplitude data of each cell were normalized to its cell capacitance (current density, pA/pF). Current-voltage relationship (I-V curve) was presented by the currents normalized by the peak currents. Voltage-dependent activation and steady-state inactivation profiles were calculated by Boltzmann fitting function.

### Recording solution

For AP, normal Tyrode's solution was used (in mM): NaCl 125, KCl 4.5, NaH_2_PO_4_ 1.8, NaHCO_3_ 24, CaCl_2_ 1.8, MgCl_2_ 0.5, and Glucose 5.5 and with pH adjusted to 7.40 with 1.0 N NaOH; and the pipette solution contained (in mM): K-glutamate 130; KCl 15; NaCl 5.0; Mg-ATP 5; MgCl_2_ 1.0; EGTA 5.0; CaCl_2_ 1.0; HEPES 10, and pH adjusted to 7.2 with KOH.

For *I*_Na_, pipette solution was (in mM): CsOH 125; Aspartic acid 35; tetraethylammonium chloride 30; HEPES 11; Mg-ATP 5.0; EGTA 10; phosphocreatine 3.6, pH adjusted to 7.30 with 1.0 N CsOH, and recording solution contained (in mM): NaCl 50; MgCl_2_-6H_2_O 1.2; CaCl_2_ 1.8; Tetraethylammonium chloride 125; CsCl 5.0; HEPES 20; Glucose 11; 4-AP 3.0; MnCl_2_ 2.0; and pH adjusted to 7.30 with 1.0 N CsOH.

For *I*_Ca_, pipette solution was prepared (in mM): CsCI 20, MgCl_2_-6H_2_O 1, Mg-ATP 5, EGTA 10, CSOH 110, asparate 110, HEPES 10, and pH adjusted to 7.2 using CsOH, and recording solution contained (in mM): Tris-Cl 136, CsCl 5.4, CaCl_2_ 2.0, MgCl_2_-6H_2_O 1.0, HEPES 10, glucose 5.0, and pH adjusted to 7.4 using Tris.

For *I*_K_, pipette solution contained (in mM): K-glutamate 130; KCl 15; NaCl 5.0; Mg-ATP 5.0; MgCl_2_ 1.0; EGTA 5.0; CaCl_2_ 1.0; HEPES 10, and pH adjusted to 7.2 with 1.0 N KOH, and recording solution contained (in mM): NaCl 138, KCl 5.4, CaCl_2_ 1.8, MgCl_2_ 1.0, CdCl_2_ 0.3, Nifedipine 0.02, HEPES 10, 10 glucose, and pH adjusted to 7.4 with 1.0 N NaOH.

### Statistical analysis

Data were collected using Clampfit and analyzed using Origin and Excel. The EC_50_ was estimated using sigmoidal fitting function from the dose-response curve. Continuous variables are presented as mean ± SD. Student's *t*-tests were used to compare the means between groups. *P* ≤ 0.05 was considered statistically significant.

## RESULTS

APs, *I*_Na_, *I*_K1_, *I*_to_, and *I*_Ca_ before and after treatments with dobutamine were investigated, respectively, in ventricular cardiomyocytes and only those completely recordings with control and test were included in the pooled data for further analysis. The number of observation in each group was collected from at least 3 mouse heart.

### Changes in AP discharge profiles in the presence of dobutamine

In isolated ventricular cardiomyocyte (Figure 1A, *n* = 12), AP discharge parameters were altered by dobutamine in a concentration-dependent manner (Figure 1B–1D, Table 1), manifested as slight but significant increase in the resting membrane potential (RMP) and AP firing threshold (APFT) toward the hyperpolarized direction, also increase in the rate of depolarization revealed by maximal upstroke velocity (UV_MAX_) measured from the derivative current changes over the membrane potential without altering the peak of APs. Whereas, AP durations (APD_50_ and APD_90_) were prolonged with decrease in the maximal downstroke velocity (DV_MAX_). These results suggest that multiple ion channel mechanisms may be involved in the changes in AP discharge profiles. Additionally, the EC_50_ of dobutamine for UV_MAX_ and APD_50_, a parameter to represent depolarization and repolarization function, were estimated differently at 0.151 μM and 0.248 μM, respectively, indicating separate mechanisms being involved in the corresponding changes. Intriguingly, the ratio of APD_50_/APD_90_ at all concentrations are nearly one (0.98∼0.99), implying that the absolute effective refractory period (ERP) was lengthened in parallel along with APD prolongation.

**Table 1:** Effects of dobutamine (0.1, 0.3, and 1 μM) on discharge profiles of AP recorded from ventricular cardiomyocytes isolated from adult mouse heart Averaged data were presented as mean ± SD, *n* = 12 myocytes from at least 4 mice. *P < 0.05 and **P < 0.01 vs control.

Parameter	Control	0.1 μM Dob	0.3 μM Dob	1 μM Dob	Washout
**RMP**	−79.4 ± 3.88	−81.2 ± 3.47	−83.6 ± 3.12*	−84.8 ± 3.44**	−80.6 ± 3.73
**APFT**	−57.1 ± 4.13	−58.8 ± 3.42	−61.3 ± 4.05*	−63.6 ± 4.66**	−58.8 ± 3.99
**Peak_AP_**	56.1 ± 5.11	56.9 ± 4.23	57.8 ± 3.41	58.8 ± 3.18	58.2 ± 4.15
**APD_50_**	3.87 ± 0.67	4.45 ± 0.81	5.51 ± 0.76*	6.14 ± 0.64**	4.33 ± 0.59
**% of APD_50_**	100%	114.98%	142.38%	158.66%	111.89
**APD_90_**	11.1 ± 2.04	13.0 ± 2.67	15.9 ± 3.82*	17.7 ± 4.37**	12.6 ± 4.02
**% of APD_90_**	100%	117.11	143.24%	159.46%	113.51%
**UV_MAX_**	193 ± 20	237 ± 14*	260 ± 17**	266 ± 27**	208 ± 15
**DV_MAX_**	−28.3 ± 4.24	−31.3 ± 4.36	−37.9 ± 3.24*	−46.4 ± 3.88**	−33.6 ± 3.78

Note: **RMP**: resting membrane potential (mV); **APFT**: AP firing threshold (mV); **Peak_AP_**: the peak of AP (mV); **APD_50_**: AP duration measured at 50% of the height (ms); **APD_90_**: AP duration measured at 90% of the height (ms); **UV_MAX_**: the maximal upstroke velocity of depolarization (mV/ms); **DV_MAX_**: the maximal downstroke velocity of repolarization (mV/ms).

### Effect of dobutamine on voltage-gated Na^+^ channel activity

**Figure 2:**
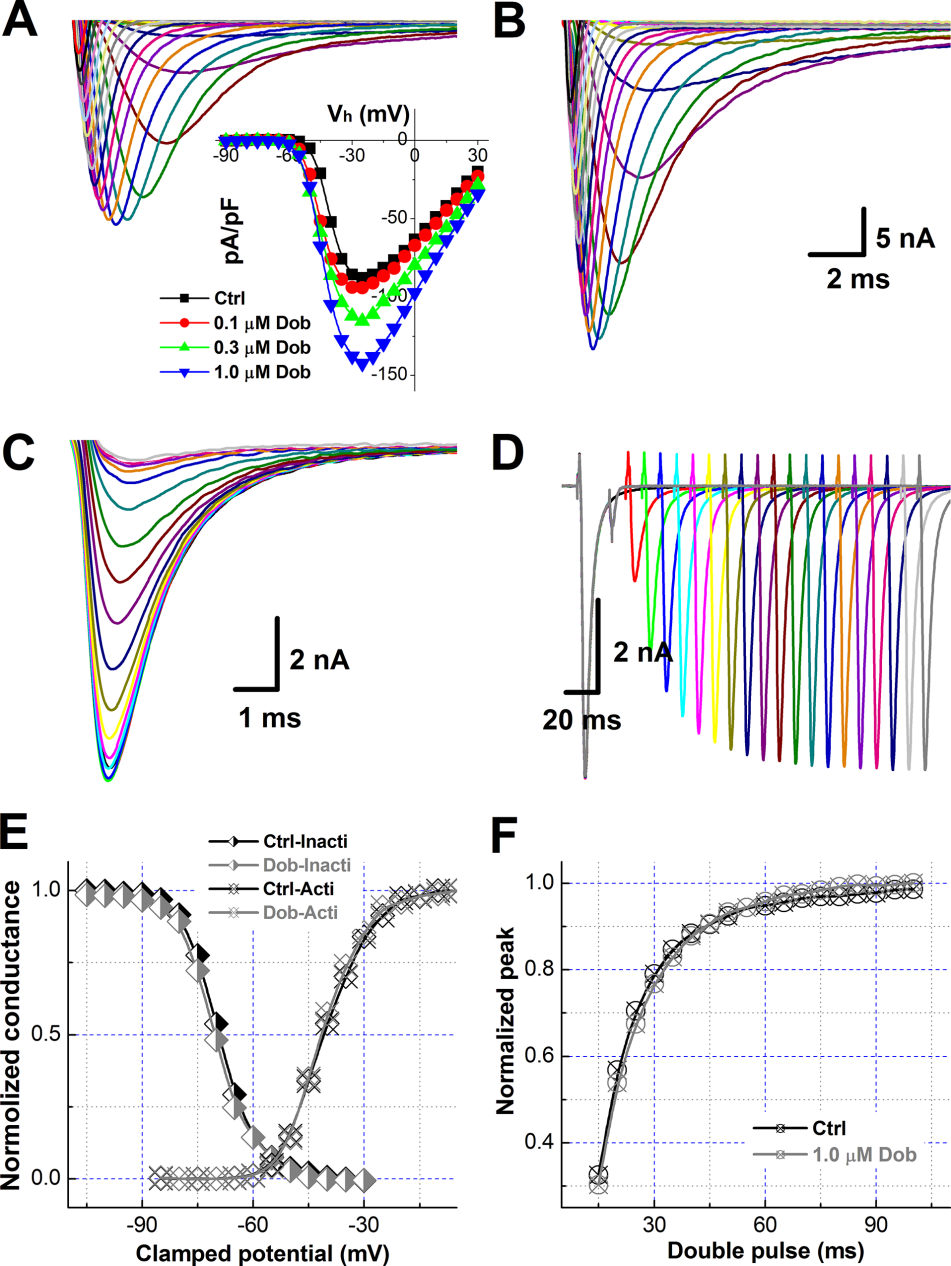
Dobutamine-mediated increase in the current density of voltage-gated Na^+^ channel (*I*_Na_) without changing the voltage-dependent properties of activation and inactivation in ventricular cardiomyocyte isolated from adult mouse heart For activation of *I*_Na_ recording in voltage-clamp mode, the cell was hold at −100 mV and 400 ms depolarization current pulse was stepped from −90 mV to +30 mV with 5 mV increments and 1-s interval between steps, for inactivation protocol, the cell was hold at −100 mV with double pulse protocol, before the test pulse 1-s conditioning pre-pulse was stepped from −120 mV to −30 mV 5 mV increment and followed by 20 ms test pulse at 2 ms immediately after the prepulse and stepped to −30 mV with 3-s step interval, for reactivation protocol. The current density (pA) was normalized by whole-cell capacitance (pF) and voltage-dependent activation and inactivation curve were fitted by Boltzmann function (normalized conductance). All representative traces shown in this figure are from the same patch recordings. **A.** and **B.** voltage-dependent activation of *I*_Na_ before and after 1.0 μM dobutamine. *inset*: the current-voltage relationship (I-V curve). Scale bars in B also apply for A; **C.** and **D.** voltage dependent inactivation and reactivation, respectively; **E.** voltage-dependent activation and inactivation curves; and **F.** voltage-dependent reactivation curves. Voltage-dependent property was fitted using Boltzmann equation and averaged data were presented as mean ± SD, *n* = 8 recordings from at least 3 mouse heart.

Upon the effect of dobutamine on the parameters of AP depolarization including RMP, APFT, and UV_MAX_, the changes in current density and/or the voltage-dependent property of Na^+^ channel in the presence of dobutamine are highly expected. In this regard, *I*_Na_ was collected (Figure 2A & 2B) and averaged data showed that the *I*_Na_ density was enhanced concentration-dependently without shifting in the peak of *I*_Na_ (Figure 2A, *inset*), however, voltage-dependent steady-status activation and inactivation, as well as reactivation profiles were not markedly changed (Figure 2C–2D) by dobutamine. The EC_50_ of dobutamine for the density of *I*_Na_ was estimated at 0.143 μM, which is correspondent well with the EC_50_ for UV_MAX_ of AP simply because the *I*_Na_ is the current underlying the AP depolarization.

### Contribution of *I*_Ca_ on dobutamine-mediated APD prolongation

**Figure 3:**
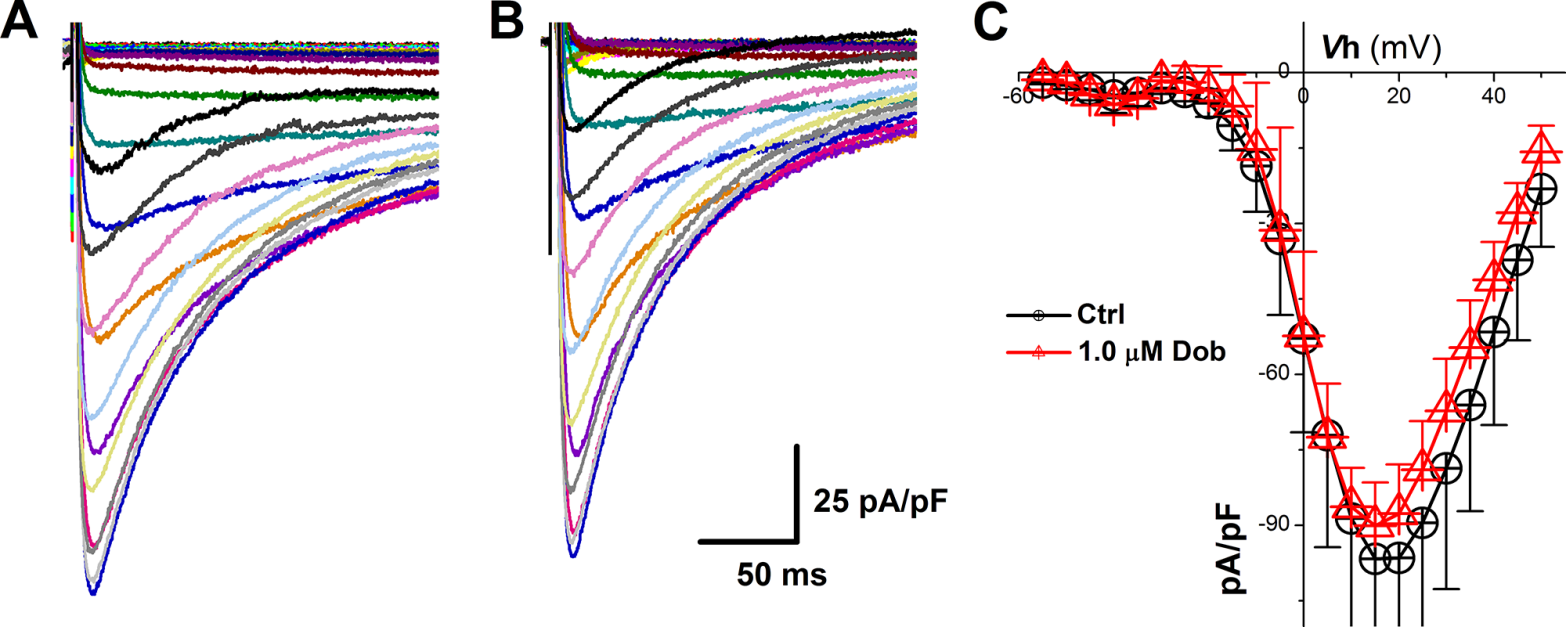
Effects of dobutamine on voltage-gated Ca^2+^ channel (*I*_Ca_) recorded in ventricular cardiomyocyte isolated from adult mouse heart For *I*_Ca_ recording, the cell was hold at −80 mV and 400 ms depolarization current pulse was stepped from −60 mV to + 60 mV with 5 mV increment and 1-s step interval. **A.** and **B.** representative recordings of *I*_Ca_ before and after 1 μM dobutamine, respectively; **C.** current-voltage relationship (I-V curve). averaged data were presented as mean ± SD, *n* = 6 recordings from 3 mouse heart.

According to lengthened APD and slowed repolarization in the presence of dobutamine, the modulation of voltage-gated Ca^2+^ channel would be one of potential mechanism. To test this hypothesis, *I*_Ca_ was recorded from ventricular cardiomyocytes isolated from adult mouse heart before and after dobutamine and the results showed that neither the current density nor I-V relationship (Figure 3) were changed by dobutamine, implying that voltage-gated K^+^ channels (*I*_to_ and *I*_K1_) would be the next target to elucidate.

### Involvement of *I*_K1_ and *I*_to_ in dobutamine-mediated slow repolarization

**Figure 4:**
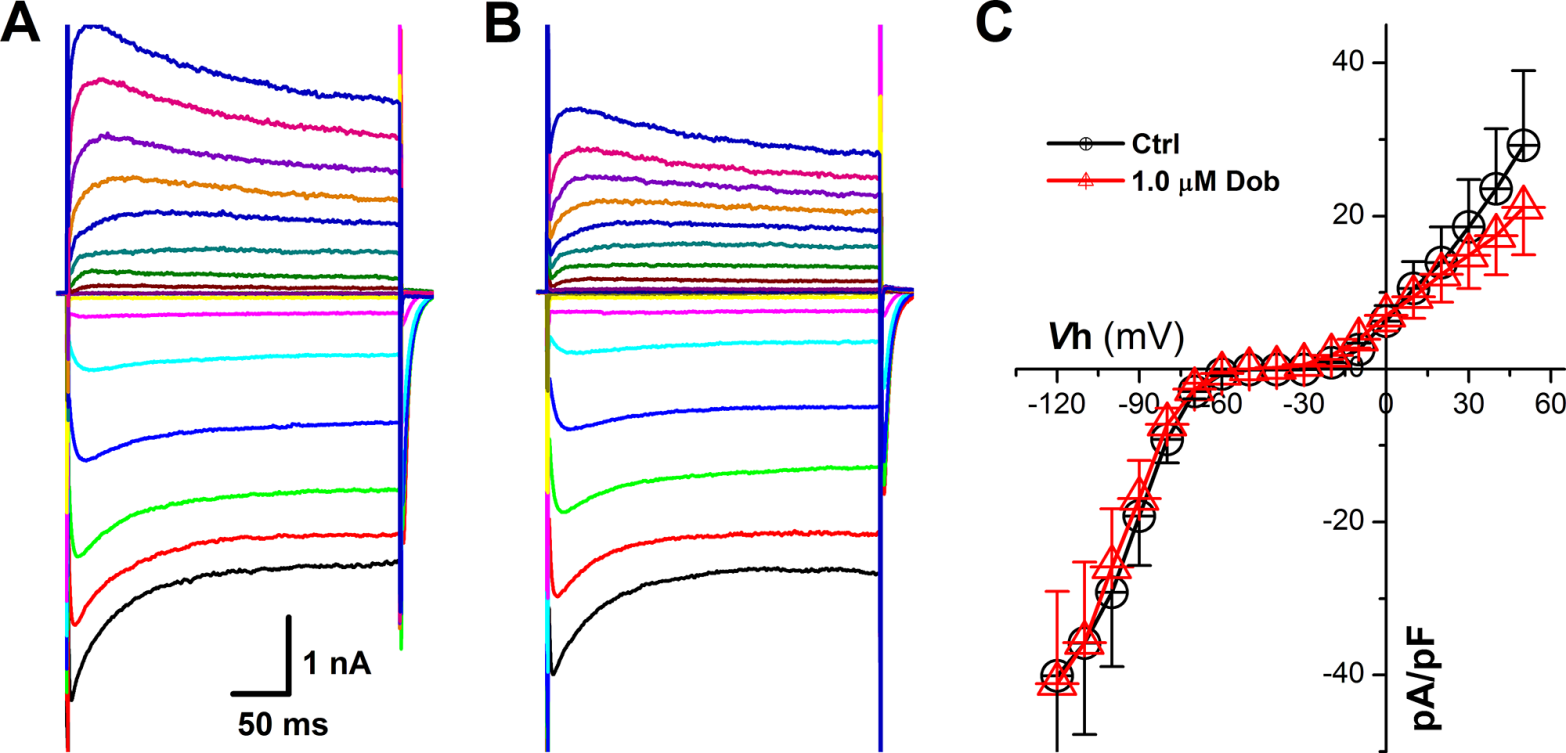
Effects of dobutamine on voltage-gated K^+^ channel (*I*_to_) recorded in ventricular cardiomyocyte isolated from adult mouse heart For *I*_to_, the cell was hold at −40 mV and 600 ms depolarization current pulse was stepped from −40 mV to + 50 mV with 10 mV increment and 1-s step interval. **A.** and **B.** representative recordings of *I*_to_ before and after 1 μM dobutamine, respectively; **C.** current-voltage relationship (I-V curve). averaged data were presented as mean ± SD.

**Figure 5:**
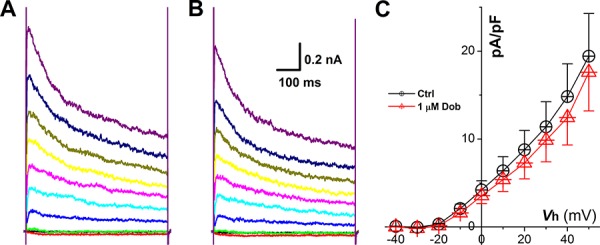
Effects of dobutamine on voltage-gated K^+^ channel (*I*_K1_) recorded in ventricular cardiomyocyte isolated from adult mouse heart For *I*_K1_, the cell was hold at −40 mV and 300 ms depolarization current pulse was stepped from −120 mV to + 50 mV with 10 mV increment and 1-s step interval. **A.** and **B.** representative recordings of *I*_K1_ before and after 1 μM dobutamine, respectively; **C.** current-voltage relationship (I-V curve). averaged data were presented as mean ± SD.

Voltage-gate K^+^ channels, such as *I*_K1_ and *I*_to_, are dominate currents for AP repolarization in mouse, which play an opposite role against *I*_Ca_, and the down-regulation of *I*_K1_ and *I*_to_ would also cause increased APD and slowed repolarization. Surprisingly, the current density and voltage-dependent property (Figure 4 & 5) for both *I*_K1_ and *I*_to_ were not modified by the highest concentration (1 μM) of dobutamine.

### Extracellular-dependency of dobutamine-mediated changes in AP repolarization

**Figure 6:**
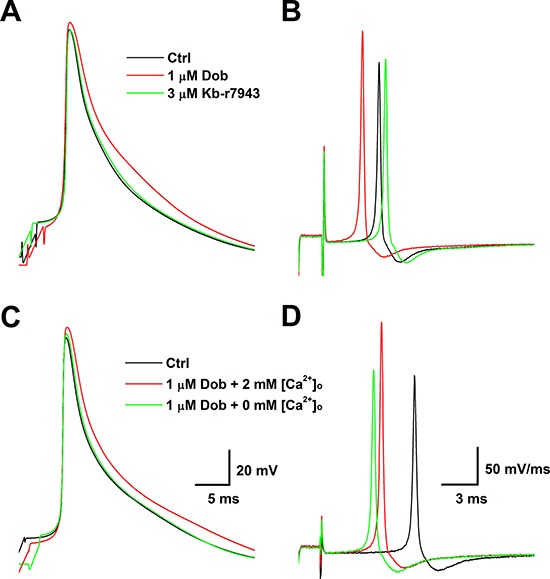
Reverse mode of Na^+^-Ca^2+^ exchanger (NCX)- and extracellular Ca^2+^ ([Ca^2+^]_o_)-dependent effects of dobutamine on AP discharge profiles **A.** and **B.** APs and derivatives before and after 1 μM dobutamine and dobutamine plus 3 μM Kb-r7943; **C.** and **D.** APs and derivatives before and after 1 μM dobutamine with 2 mM [Ca^2+^]_o_ and dobutamine with 0 mM [Ca^2+^]_o_. Scale bars shown in C and D are also apply for A and B, respectively.

Even though, *I*_K1_ and *I*_to_, and *I*_Ca_ as well were not involved in dobutamine-mediated AP repolarization changes, intracellular Ca^2+^ mobilization through other transmembrane mechanism rather than voltage-gated Ca^2+^ is then expected. To verify if the extracellular Ca^2+^ influx occurs during the AP repolarization, removing extracellular Ca^2+^ instead of using Mg^2+^ would be the easiest way. In another set of experiment, the effect of dobutamine on AP was repeated in the normal recording solution with 2 mM Ca^2+^. Interestingly, the similar results induced by 1 μM dobutamine were disappeared at ∼2 min after complete bath perfusion (∼1 ml/min) with 0 mM Ca^2+^ recording solution (Figure 6A & 6B), strongly indicating the Ca^2+^-dependency of dobutamine-mediated changes in AP repolarization.

### Effect of Kb-r7943 on dobutamine-mediated changes in AP repolarization

Recent observation provides an evidence showing the potential connection between Nav1.5 and Na^+^-Ca^2+^ exchanger (NCX) [3, 4], which is a possible clue for an explanation regarding the effects of dobutamine AP discharge profiles. To test this hypothesis in our experimental condition, the APs were elicited in current-clamp configuration in ventricular cardiomyocytes. The results showed that the AP repolarization changes induced by 1.0 μM dobutamine were completely blocked by the pretreatment of cardiomyocyte with 3 μM Kb-r7943 (Figure 6C & 6D), a selective blocker for NCX, without affecting increased AP depolarization (UV_MAX_).

## DISCUSSION

Dobutamine is a sympathomimetics and activates β-adrenergic receptors (β_1_ and β_2_ mediating cardiac and vascular effects, respectively) as an inodilator [12] and can be used in cases of congestive heart failure to increase cardiac output and positive inotropic support in the short-term treatment of patients with cardiac decompensation due to depressed contractility. Even though the clear beneficial effect of dobutamine the appropriate role of intravenous inodilator therapy in the management of congestive heart failure has long been a subject of controversy and limitation, mainly because of the side effects such as increased heart rate and O_2_ consumption via β-receptor activation [13], and direct vascular effect and baroreflex feed-back regulation [14]. So, further investigation is definitely necessary to elucidate the exist electrophysiological mechanism underlying the therapeutic effect of dobutamine, which may in turn benefit for the future clinical application and pharmacological convention of dobutamine.

The major finding of this observation has demonstrated, for the first time by our knowledge, that the extracellular Ca^2+^-dependent Na^+^-induced Ca^2+^ influx is confirmed in ventricular cardiomyocyte in the presence of dobutamine in a concentration-dependent manner through voltage-gated Na^+^ channel (Nav1.5) activation during AP depolarization and consequently activation of the reverse mode of NCX and the intracellular Ca^2+^ mobilization during prolonged AP repolarization, which may be a novel mechanism of positive inotropic action of dobutamine except for the known β_1_-receptor activation and G-protein coupled cAMP pathway.

Clearly, dobutamine accelerates the AP depolarization with shifting of RMP and AP firing threshold toward the hyperpolarized direction and this observation is supported by the notion of increased *I*_Na_ density presumably due to the enhanced availability of voltage-gated Na^+^ channel at given relatively lower potential upon the inactivation profiles. In this particular case, a relatively less energy would be required to charge the membrane and therefore causing the reduction of AP firing threshold without alternation of voltage-dependent activation and inactivation properties. However, dobutamine-induced RMP hyperpolarization might be explained by the dobutamine-induced myocardial K^+^ uptake by β_1_-adrenoreceptor and adenylate cyclase activation [13] and the Nernst Equation.

Not surprisingly, the outward K^+^ currents are the first to respond to a depolarizing event on account of the hyperpolarized activation profile and fast activation time constant for these currents. Moreover, as the membrane potential enters into a phase of rapid depolarization there is a marked recruitment of transient and Ca^2+^-activated K^+^ currents, which are primary outward K^+^ currents responsible for the terminating the AP upstroke and initiating a reversal in the trajectory of the membrane potential upon a Hodgkin-Huxley model [15–17]. In other words, the more faster depolarization occurs, the more K^+^ channels would be recruited during the repolarization and sequentially causing a shorter AP duration. Interestingly, although dobutamine caused a fast depolarization the larger K^+^ currents recruitment, especially the transient (*I*_to_), were not observed from the voltage-clamp records, suggesting other ion channel mechanism being involved in this event and the most suspicious one is *I*_Ca_, unfortunately, this hypothesis was also not confirmed under the current experimental condition.

Recently, several reports indicate the potential role of the reverse mode of NCX in an intracellular Ca^2+^ mobilization [3, 4], which could be cytoplasmic Na^+^-dependent [5] and activated during the AP depolarization [18] in mouse and human. In this regard, by preincubation of ventricular cardiomyocytes with Kb-r7943, a selective NCX blocker, dobutamine-mediated changes in repolarization were disappeared without altering the depolarization, which was also confirmed by simply removing the extracellular Ca^2+^. Even though the extracellular Ca^2+^ was not added in our recording solution, the concentration for extracellular Ca^2+^ came from the distilled water and chemicals would be close to 1 μM [19], which may be enough to maintain the cell function and signaling cascades. These data strongly suggest that dobutamine causes extracellular Ca^2+^-dependently Na^+^-induced Ca^2+^ influx through the reverse mode of NCX.

Collectively, in the presence of dobutamine, unchanged ratio of APD_50_/APD_90_ may not increases the risk for arrhythmogenesis because the effective refractory period remains no change along with the similar extend prolongation of both APD_50_ and APD_90_. Meanwhile, the prolonged AP duration would limit heart rate increased by β_1_-receptor stimulation, fast depolarization would produce a fast ventricular contraction and offer a relative longer period of time for cardiac diastolation. KB-R7943 is a potent, selective inhibitor of the reverse mode of the Na^+^/Ca^2+^ exchanger, but its modulatory effects on other receptor systems, such as the inhibition of Kb-r7943 on nicotinic receptor [20] and N-methyl-D-aspartate receptor [8] in nervous system, can not be excluded in cardiomyocytes under the current investigation. It would be necessary to verify the effect of Kb-r7943 on β-adrenoreceptor in the future experiment although it has not been documented so far.

## Abbreviations

NCX: Na^+^/Ca^2+^ exchanger
